# Regulating the health workforce in Europe: implications of the COVID-19 pandemic

**DOI:** 10.1186/s12960-021-00624-w

**Published:** 2021-07-10

**Authors:** Dimitra Panteli, Claudia B. Maier

**Affiliations:** 1 Department of Health Care Management, Technische Universität Berlin, Strasse des 17. Juni 135, 10623 Berlin, Germany; 2 European Observatory On Health Systems and Policies, Place Victor Horta 10/30, 1060 Brussels, Belgium

**Keywords:** Health professional regulation, European Union, Cross-country collaboration, COVID-19 pandemic

## Abstract

In the European free movement zone, various mechanisms aim to harmonize how the competence of physicians and nurses is developed and maintained to facilitate the cross-country movement of professionals. This commentary addresses these mechanisms and discusses their implications during the COVID-19 pandemic, drawing lessons for future policy. It argues that EU-wide regulatory mechanisms should be reviewed to ensure that they provide an adequate foundation for determining competence and enabling health workforce flexibility during health system shocks. Currently, EU regulation focuses on the automatic recognition of the primary education of physicians and nurses. New, flexible mechanisms should be developed for specializations, such as intensive or emergency care. Documenting new skills, such as the ones acquired during rapid training in the pandemic, in a manner that is comparable across countries should be explored, both for usual practice and in light of outbreak preparedness. Initiatives to strengthen continuing education and professional development should be supported further. Funding under the EU4Health programme should be dedicated to this endeavour, along with revisiting the scope of necessary skills following the experience of COVID-19. Mechanisms for cross-country sharing of information on violations of good practice standards should be maintained and strengthened to enable agile reactions when the need for professional mobility becomes urgent.

## Main text

The COVID-19 pandemic clearly demonstrated the importance of ensuring adequate numbers and a balanced skill-mix of health professionals, as well as the necessity for an overview of how professional competence is developed, maintained and demonstrated across countries. Health professional regulation is essential for setting the framework within which this can be achieved, end encompasses laws or bylaws defining the minimum requirements for education, entry to practice, title protection, scope-of-practice, continuing professional development and sanctioning. In the European Union and European Economic Area (EU/EEA), the free movement of citizens and the uneven distribution of staff shortages had necessitated regulatory action to enable health professional migration long before the COVID-19 pandemic. However, its scope does not capture the full range of measures necessary to ensure health professionals acquire and maintain competence throughout their careers.

There is substantial variability of country-level practices on workforce regulation in Europe; this reflects the reality that countries are generally free to decide the extent and type of regulatory mechanisms they want to apply to the health professions. These span command and control, meta-regulation, self-regulation, and market mechanisms. The chosen constellation usually relates to the complexity of the professional’s role and its implications for patient safety.

**Fig. 1:**
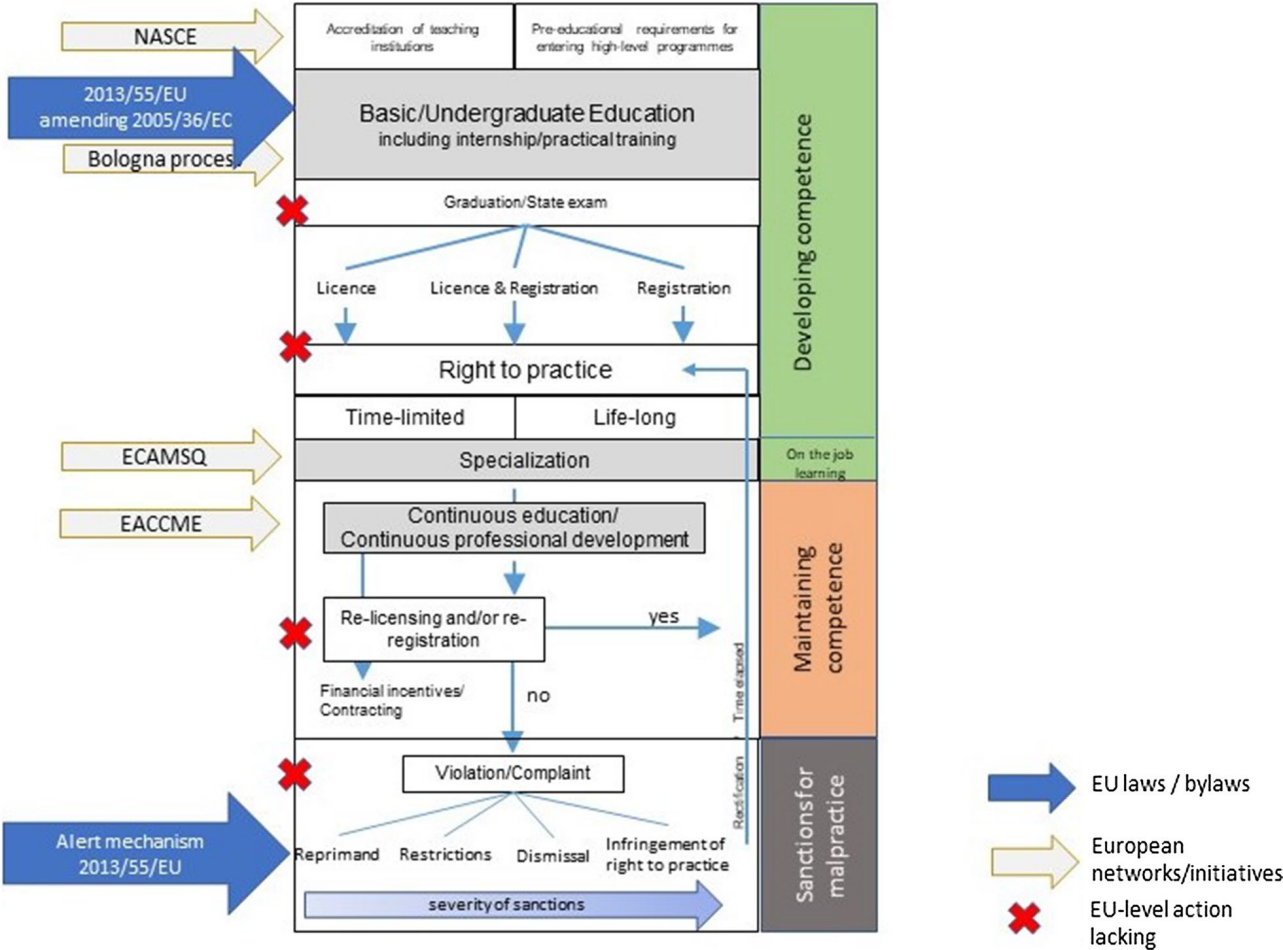
EU action along the pathway of professional competence development and upkeep. Source: modified from [1]

In this contribution, we focus on highlighting how initiatives at the European level have contributed towards a more harmonized understanding of minimum requirements for physicians and nurses, and where there still might be room for action. We follow the logic of the adapted framework for strategies to regulate health professionals shown in Fig. 1, which draws on previous work on effectiveness and implementation of different strategies to regulate health professionals [1]. Finally, we highlight the implications of the COVID-19 pandemic for future directions in relevant health professional regulation policy along these steps.

## Health professional education and training: EU regulation focuses on the primary educational qualifications, but new, flexible regulatory mechanisms are required for specializations

Arguably, the cornerstones of building competence for health professionals are education and training. Country practices vary starting from entry requirements for higher education, for which there is no standardization at EU level. In Europe, national authorities are responsible for the recognition of health education institutions (e.g. via accreditation) and educators in their jurisdiction. For medical education specifically, the European Union of Medical Specialists (Union Europeene des Medecins Specialistes, UEMS) established the Network of Accredited Skills Centres in Europe (NASCE). NASCE evaluates and subsequently accredits institutions of medical education and training in European countries, but this pertains to particular skill sets rather than entire curricula of basic medical education [2].

The regulation of curricula for the health professions at the national level usually aims to ensure uniformity across educational programmes. National regulations for the basic curricula of professional education in Europe and the European Economic Area are determined by the EU Directives on the mutual recognition of professional qualifications. Directive 2013/55/EU of 20 November 2013 amending Directive 2005/36/EC of the European Parliament and of the Council of 7 September 2005 set out the legal foundation to ensure that health professionals can move freely and practise across Member States. The EU Directives mainly regulate the minimum duration of training to ensure comparability and equivalence of diplomas, but not the details of its content. For instance, medical education requires a minimum of 5 years of university-based theoretical and practical training, but the detailed composition and set-up beyond this remains a national (or sub-national) responsibility. Starting in 1999, the Bologna process has aimed to enhance the comparability of higher-education qualifications in Europe and ensure their quality. It applies to educational programmes for health professionals and has drastically influenced the way they are organized.

Physicians usually complete studies in medicine at university level and require (additional) training at a hospital in order to obtain a medical degree and be able to practice. They then undergo specialist training mostly by means of on-the job learning; completing specialist training is usually a prerequisite to deliver patient care independently. In contrast to the approach towards basic education described above, self-regulation plays the main role for determining the requirements for specialist training or residency programmes. This leads to considerable variability across European countries regarding admission policy, duration, scope, terminology and significance of diplomas, and general structure of residency training. The EU Directives primarily list the titles and minimum duration of specialist training for physicians in EU countries. The UEMS, founded in 1958, is the representative organization of all medical specialists in the European Community, aiming to encourage the harmonization of specialist training across Europe. Already in the 1990s, it issued guiding principles for a European approach for specialist medical training to ensure quality and comparability, meant to guide but not replace existing national structures. The UEMS also established the European Council for Accreditation of Medical Specialist Qualifications (ECAMSQ®), which developed a voluntary competence-based framework for the assessment and certification of medical specialists in Europe [3]. By virtue of their voluntary nature, these initiatives are primarily of value when they are recognized by the relevant national bodies in each country.

Compared to medical education, the regulation of nursing education is even less uniform across countries and can apply at national or sub-national level. The European Directives mandate a minimum requirement of 4.600 h theory and practice (Directive 55/2013/EU) and certain skills and competencies which need to be obtained by nurses (e.g. ascertaining the need for nursing care, to plan, organize and implement nursing care, to empower individuals and patients), but the contents of curricula remain highly heterogeneous across countries. As a result of the Bologna process, nursing education is performed increasingly at higher educational institutions (via Bachelor and Master programmes), but primary education in nursing schools also co-exists in many countries. This shift towards degree-level nursing education paved the way for advanced practice and the expansion of professional roles for nurses. Some form of specialized training is available in most European countries, albeit with varying titles, levels and length of education. Depending on the type and level of education and specialization, professionals are qualified to take over different tasks and responsibilities. This has implications for the regulation of task and responsibility division between professions and the complexity of ensuring comparability for professionals moving across borders.

European countries have different requirements for granting the right to practice for both physicians and nurses, but the successful completion of basic professional education is the minimum. The successful completion of an examination is usually required, and this can be integrated in the prerequisites to obtain the academic degree or be additional. Obtaining a license to practice is often linked to obligatory registration in a health professional register, which aims to inform the public and potential employers about the professional’s qualifications. Such registries are operated in most European countries for physicians, while only few have a nurse registry in place. Licensing and registration for doctors and nurses are mostly regulated at national or sub-national level. Competent bodies vary from governmental ministries to self-regulating professional bodies, with varying degrees of statutory control and professional associations playing a key role in most countries [1]. There is no EU-wide licensing authority or registry for either physicians or nurses.

## Keeping up with best practice: harmonizing continuing education

Over the course of their career, health professionals must constantly maintain and update their skills to be able to provide safe and effective patient care in line with best available knowledge. In Europe, few countries rely on the responsibility of professionals themselves to ensure that they remain fit to practice over time [1]. Most have introduced formal mechanisms to demonstrate continued professional competence, such as mandatory continuing education (or continuing professional development, see [1] for disambiguation), mandatory re-licensing, peer review and external inspection. Continuing professional education and development are used increasingly in European countries. However, the definition of formal standards that ensure related activities are effective and go beyond being a bureaucratic requirement remains a challenge. The configuration of processes to demonstrate continued competence in European countries varies considerably, along with the content and duration of eligible courses. For both physicians and nurses, it is the professional associations who decide which activities get accredited and monitor the participation of their members; the prerequisites to demonstrate sustained competence are generally more regulated for physicians than for nurses. There is no European-level regulatory harmonization for either profession.

However, in 2000 the UEMS established the European Accreditation Council for Continuing Medical Education (EACCME®) to encourage high standards in the development and delivery of continuing medical education and professional development courses and foster harmonization. The purpose of the EACCME® is to accredit courses and facilitate the recognition of credits between countries based on cooperation agreements with countries in Europe and beyond [4]. Twenty European countries and one Italian region have signed such agreements; other countries can also recognize EACCME® credits voluntarily, but professionals need to go through the national or sub-national competent authorities to ensure this happens. In response to criticisms about the content and independence of continuing education and professional development courses from commercial interests, the EACCME® developed quality criteria on which their accreditation process is based.

## A cross-country alert mechanism for violations of good professional practice

Incidents indicating the violation of good professional practice are handled in various ways in European countries. Possible actions encompass reprimands and financial penalties, the temporary or permanent withdrawal of the right to practice and/or shortening of the registration period in systems where the bestowment of the right to practice is time-limited. Complaints about the practice of a health professional are brought to the attention of competent regulatory authorities by patients and their relatives, employers with monitoring tasks or specific organizations for the oversight of the health professions. Disciplinary action in which the professional’s competence to practice is investigated can be handled internally or lead to civil litigation proceedings. In more serious cases that go beyond the violation of good practice and professional codes of conduct, such as criminal offences, the corresponding legal pathways are followed [1].

The increasing mobility of health professionals created a necessity for a better common understanding and reporting of such incidents across countries. Before 2016, health professionals who had been sanctioned in one country could move to another and continue practising without consequences, and such cases have been reported. Directive 2013/55/EU on the mutual recognition of professional qualifications established an alert mechanism to flag (even temporary) bans or restricted from practice, and enable warnings across Member States. The evaluation of the first two years of functioning of this mechanism was favourable, but highlighted the need for its continuous monitoring and adaptation. It revealed that more than 20,000 alerts were sent by competent Member State authorities, mostly pertaining to cases of professionals who were restricted or prohibited from practice [5].

## Implications and lessons learned from the COVID-19 pandemic

During the COVID-19 pandemic most countries in Europe have used a variety of different strategies to upskill and re-deploy their existing health workforce [6]. Examples include expanding the working hours of already employed physicians and nurses, additional trainings, e.g. in intensive care or emergency care, or attracting medical and nursing students in their final year of studies to work. Some countries recruited retirees back into active practice, hired volunteers or professionals and students from other European countries [7–9]. These strategies have had implications on the skill-mix of the workforce, competencies and teamwork. Most of the strategies are not regulated and were applied ad hoc. In Germany, for instance, the procedures for mutual recognition of diplomas of EU-trained health professionals were sped up. In contrast, lengthier procedures are in place for specialist training, e.g. for nurses, which is not automatically recognized. At EU level, more flexible mechanisms to recognize the specializations of physicians and nurses, for instance in intensive care or emergency care, would solve this problem and equip European health systems for more expedient responses in the future.

Furthermore, rapid training of health professionals to obtain new skills, for instance in intensive care, took place in many European countries. Hospitals in several countries, including Germany, introduced rapid training courses for nurses to use ventilators or extracorporeal membrane oxygenation (ECMO) machines in intensive care units (ICU). The skills obtained should be documented not only nationally, but also at European level, to enable the rapid identification of appropriate personnel in times of urgent need. For the same purpose, physicians and nurses and other health professionals with expertise in treating COVID-19 patients, for instance in highly specialized centres, should be easily identifiable across Europe. Supporting and expanding relevant networks would help to strengthen the sharing of expertise and skills. Health professionals should be able to document these skills, for instance via the European Skills Passport [10]. A more formalized option could include a (voluntary) note or annotation in professional registries, but this would require a network of EU-wide registries and can be considered a mid- or long-term initiative.

Alert mechanisms on violations of good professional practice are of high relevance, also during the pandemic. While the vast majority of health professionals have worked effortlessly and to highest professional standards to treat patients under time pressure, the risk of transgressions remains. Hence, relevant occurrences should be identified and shared across European countries. At the same time, countries need to identify legal protection mechanisms for physicians, nurses and other health professionals who have undergone rapid training, to ensure the newly acquired skills are legally protected. Furthermore, the recruitment of retired health professionals or medical and nursing students poses new challenges from a regulatory perspective, as they are not covered under the usual provisions for health professionals and require new supervisory structures as well as legal and regulatory mechanisms. Lessons on how to deal with these new members of teams should be shared across countries.

## Moving forward: priority areas for cross-country collaboration

We have previously argued that the regulation of health professionals should be viewed in a holistic manner, taking all strategic components shown in Fig. 1 into account, with the aim of creating learning systems of regulation that combine effective checks and balances with a flexible response to global needs for a competent, sufficient workforce [1]. The reasonableness of such an approach was only underlined during the COVID-19 pandemic, and the resulting necessity for surging appropriately skilled health workforce capacities to meet health care needs.

The EU Directives have provided a relative basis for standardization of health professional education for physicians and nurses, and the Bologna process has contributed substantially to comparability in the EU/EEA space. While curricular set-up and content still varies across countries, this is not necessarily disadvantageous: too restrictive regulation can hamper innovation in curricular design; encouraging exchange by supporting existing and future platforms to enable cross-country learning is important.

Increased professional mobility, staff shortages and political developments put the application of the free movement of citizens within Europe on the agenda even before the pandemic; since the emergence of COVID-19 the need for swift action to surge workforce capacities made the necessity for a common understanding of professional competence beyond basic educational requirements even clearer. Accordingly, detailed work on the status quo of cross-country variation in the specializations of physicians and nurses would help develop more flexible mechanisms for recognition and professional movement. Documenting new skills in a manner that is understandable and transferrable across countries should be explored, both for usual practice and in light of outbreak preparedness. At the same time, it is important to consider that an increased dependence on foreign healthcare workers can negatively impact staff availability in their countries of origin. It is therefore vital that new mechanisms to enhance health system resilience are developed with an international perspective, and account for the need to maintain necessary workforce levels across countries.

The variability of requirements for continued education and professional development for both physicians and nurses can hamper cross-country movement for temporary and long-term professional mobility. Collaborative initiatives at European level, such as the accreditation of courses by the EACCME, should be strengthened and expanded. Considering the lack of evidence on the effectiveness of different regulatory set-ups for maintaining competence, the mixed evidence on the usefulness of different learning modalities, as well as the need to revisit the scope of necessary skills following the experience of COVID-19, funding under the EU4Health programme should be made available to strengthen relevant research.

Finally, the European alert mechanism for infringements against good professional practice constitutes an important milestone towards ensuring safe and effective patient care in light of professional mobility within the European Union. However, its evaluation and potential adaptation over time should not be overlooked, particularly given potential developments in country-level practices as systems of redress evolve over time. Accessibility and the speed with which underlying information is updated are crucial parameters to consider under the lens of swift reactions for immediate needs of professional movements across countries.

## Data Availability

Not applicable.
